# Examining the role of information exchange in residential aged care work practices-a survey of residential aged care facilities

**DOI:** 10.1186/1471-2318-12-40

**Published:** 2012-08-02

**Authors:** Sarah Gaskin, Andrew Georgiou, Donna Barton, Johanna Westbrook

**Affiliations:** 1 Centre for Health Systems and Safety Research, University of New South Wales, Kensington, Sydney, Australia; 2 Sir Moses Montefiore Jewish Home, Randwick, Sydney, Australia

**Keywords:** Informatics, Residential facilities, Long term care, Quality of care, Safety, Evaluation

## Abstract

**Background:**

The provision of residential aged care is underpinned by information, and is reliant upon systems that adequately capture and effectively utilise and communicate this information. The aim of this study was to explicate and quantify the volume and method by which information is collected, exchanged within facilities and with external providers, and retrieved from facility information systems and hospitals.

**Methods:**

A survey of staff (n = 119), including managers, health informatics officers (HIOs), quality improvement staff, registered nurses (RNs), enrolled nurses (ENs)/endorsed enrolled nurses (EENs) and assistants in nursing (AINs) was carried out in four residential aged care facilities in New South Wales and Victoria, Australia. Sites varied in size and displayed a range of information technology (IT) capabilities. The survey investigated how and by whom information is collected, retrieved and exchanged, and the frequency and amount of time devoted to these tasks. Descriptive analysis was performed using SPSS, and open responses to questions were coded into key themes.

**Results:**

Staff completed a median of six forms each, taking a median of 30 min per shift. 68.8% of staff reported transferring information from paper to a computer system, which took a median of 30 min per shift. Handover and face-to-face communication was the most frequently used form of information exchange within facilities. There was a large amount of faxing and telephone communication between facility staff and General Practitioners and community pharmacists, with staff reporting sending a median of 2 faxes to pharmacy and 1.5 faxes to General Practitioners, and initiating 2 telephone calls to pharmacies and 1.5 calls to General Practitioners per shift. Only 38.5% of respondents reported that they always had information available at the point-of-care and only 35.4% of respondents reported that they always had access to hospital stay information of residents after hospital discharge.

**Conclusions:**

This survey identified a high volume of information exchange activities, as well as inefficient procedures, such as the transfer of information from paper to computer systems and the reliance upon faxes for communication with external providers. These findings contribute to evidence for the need for interoperable IT systems to allow more efficient and reliable information exchange between facilities and external providers.

## Background

Aged care is an information intensive setting. Residential aged care facilities (RACFs) are responsible for the daily recording, maintenance and reporting of a wide range of information that relates to the administration and operation of their facility and the care of each resident [1]. RACFs require information systems and processes which are able to meet both the information needs of the organisation and a range of external stakeholders [2]. A major challenge is ensuring these systems are efficient and provide all the necessary information for supporting care and management activities. RACFs operate in environments where external information demands from funding or accreditation bodies frequently change. This requires information systems that are flexible [3]. Paper based information systems often need to incorporate new forms. Without regular review these new data collection activities can lead to the emergence of inefficient data collection processes. Further, RACFs are reliant upon a number of external health care providers and organisations to supply information [2].

Information technology has a great capacity to support efficient and effective information processing in aged care [3,4], yet its use has been limited [4-7]. One of the contributing factors has been the implementation of information systems which have largely borrowed their designs from hospital and general practice systems with limited modifications to account for the particular environment and work processes of aged care [8]. Staff may be reluctant to use IT as the systems do not easily integrate into their work processes and many remain unconvinced that they will deliver effective outcomes [7,9,10]. There is a need for the design of IT systems which are based on a detailed understanding of the work practices and requirements of RACFs. Studies investigating work practices have demonstrated that documentation consumes a large amount of staff time in aged care facilities [11,12], but more evidence is needed about the characteristics and nature of these documentation activities and how they relate to the provision of quality and safe residential care. Understanding the current information processes within RACFs is foundational to being able to design systems which support more efficient and effective work practices.

The aim of this study was to undertake a survey of four residential aged care facilities to explicate and quantify three dimensions of information exchange: 1) what information is collected and how; 2) how this information is exchanged-between staff, between documentation systems and between facility and external providers; and 3) how information is made available from hospitals and retrieved from within the facility information system.

## Methods

### Qualitative study

A qualitative study of aged care facilities was carried out prior to the survey development [1]. RACFs in NSW and Victoria were invited to participate in the study. Facilities that expressed an interest were purposively selected for the study to ensure the sample covered a range of characteristics, including facility location, facility funding model, proportion of high and low care residents, proportion and types of facility staff, as well as the facilities’ information and communication technology capacity. Overall, six facilities were selected to participate; three in NSW and three in Victoria. Four of the sites were located in metropolitan areas (outer suburban locations); one site was located in the inner city and one site was located in regional NSW. Four of the sites were not-for-profit, and two were private. Sites had a range of proportions of high and low care residents, and a mix of staff. This study, which involved interviews (n = 54) and focus groups (n = 11) of RACF staff, aimed to investigate work processes and the potential use of information technology (IT) in RACFs. The focus groups and interviews generated qualitative data that was grouped thematically into the following categories: the role of documentation within the facility, professional roles within the facility, the type of information generated, stored and exchanged in facilities, communication within and external to the facility, and the use of information and communication technologies within the facility.

### Survey development

The survey tool was developed by the research team, based on the results from the qualitative study. It aimed to quantify the time, amount, and type of information exchange processes that were described in the qualitative study. The initial survey was iteratively developed, undergoing several revisions by the research team before pilot testing in December 2010 by a group of five nurse managers from different RACFs. This pilot testing was followed by a discussion with the nurse managers who provided additional feedback as to how the survey could be improved. The final survey consisted of 35 questions; 14 of which required free text response, 6 which required the selection of responses from specified scales, and 15 multiple choice questions (refer to additional file 1 for full survey). The questions covered the recording of information in the facility, access to information, medication management processes, preparation of facility and resident related summary reports, internal staff communication, communication with the residents’ families (not reported in this paper), external communication, use of information and communication technologies in the facility and demographic questions including job title, age, years worked in current position and level of education.

### Sample

The six sites that had participated in the qualitative study were invited to participate in the survey. Facility management were given an estimate of the amount of time that survey distribution, completion and follow up would take. Four of the sites were able to provide this time and access for the research team. These facilities, located in metropolitan NSW (n = 2) and Victoria (n = 2), ranged in number of residents, proportions of high and low care residents, and number and type of staff. Managers at each of the facilities were asked to classify the extent to which specific documentation tasks within their facilities were paper-based or computerised using a five point scale which included: all paper, starting to use computers, mostly computers, nearly paperless and paperless (communicate with computers only)(Table 1). Additionally, managers were asked to classify overall which group they felt best described their facility’s documentation system.

**Table 1:** Reported use of paper and IT for different information exchanges by RACF managers

	**Facility 1**	**Facility 2**	**Facility 3**	**Facility 4**
Monitoring of resident care tasks	NA	Mostly computers	Mostly computers	Nearly paperless
Nursing information e.g. care plans	Nearly Paperless	Paperless and communicate with computers	Mostly computers	Paperless and communicate with computers
Internal communication	NA	Mostly computers	Starting to use computers	Mostly computers
Communication with GP	Starting to use computers	Starting to use computers	All paper	All paper
Communication with pharmacy	All paper	All paper	All paper	Starting to use computers
Communication with allied health	All paper	Starting to use computers	Mostly computers	Paperless and communicate with computers
Finance and administration	Nearly paperless	Starting to use computers	Mostly computers	Mostly computers
Overall documentation system	Mostly computers	Mostly computers	Mainly paper (starting to use computers)	Nearly paperless

### Participant selection and survey collection

After coordinating with facility management an information session at each site was held where the survey and participant information sheets detailing the survey were distributed to staff. Respondents filled out the survey anonymously, and placed it in an envelope which was mailed back to (or collected by) the research team. Responses were sought from all fulltime staff, comprising nurses (Registered Nurses-RNs), carers (Enrolled nurses-ENs, Endorsed Enrolled nurses or Assistants in Nursing –AINs) and managers/Health Informatics Officers (HIOs). One respondent classified themselves as having a certificate in quality improvement, this person was included in the managers and HIOs category as they are not involved in direct care of the resident. Allied health workers were not included as they are generally not employed full time at facilities. The label “AIN” was used as an umbrella term which included all types of carers who are referenced differently across Australian states. This category included personal care workers, aged care employees and care services workers.

### Ethics

The research was approved by the Human Research Ethics Committee of the University of New South Wales (HREC 10319).

### Analysis

Data were entered by a member of the research team and checked by an independent researcher. Descriptive statistics were calculated using SPSS v19. The data for all items using continuous variables were skewed, as determined by plotting the frequencies; hence the median was reported as this was considered a more appropriate measure of centrality. The mean has also been provided. One of the questions regarding the most effective method for communicating with staff within the facility had a follow up free text question which asked participants to explain why the methods they selected were most effective. Our analysis of the text identified eight categories that related to why staff preferred certain modes of communication. These categories emerged as a part of a grounded theory approach. Researcher A performed the open coding and axial coding into an initial set of categories. Agreement was sought with Researcher B as to the axial coding, and 8 codes were defined. Researcher B then re-read the open text responses and coded them into the 8 defined categories. Where there was disagreement a third researcher was consulted to obtain consensus.

## Results

### Sample

The overall response rate for the survey for nurses and carers was 52.6% (some facilities could only provide an estimate of total staff numbers) with 119 completed surveys from the four sites (Table 2). Response rates varied by professional group (Table 2). The highest response rate was for RNs (95.8%) and ENs/EENs (75%). Sixty percent of respondents were over 40 years of age (<20 years =2.6%, 20–29 years =22.6%, 30–39 years =14.8%, 40–49 years = 25.2% and ≥50 years =34.8%). The composition of workers sampled approximates that of the national composition of workers in aged care [13]. The median years of experience in current position was five years across the total sample, ranging from one month to 38 years. For RNs it was ten years, for ENs it was eight years and 9 months, and for AINs it was 4 years. Respondents were asked to report their IT skills (poor, fair, good, very good, excellent). Ninety-two (78.6%) of the 117 respondents reported having good- excellent IT skills (the remaining 19 respondents (16.2%) reported having fair skills, and 6 (5.1%) reported poor IT skills). 

**Table 2:** Site characteristics and staff response rates

**Facility**	**Location**	**Number of resident**	**Types of resident**			**Documentation system**	**Number of staff responded**			
			**High care**	**Low care**	**Respite care**		**Managers, HIO**	**RN (FTE)**	**EN/EEN (FTE)**	**AIN (FTE)**
Site 1	Suburban	78	60%	40%	0%	Mostly computers	1	2/4	0/1	13/30
Site 2	Metro	90	70%	28%	2%	Mostly computers	3	2/3	3/3	10/31
Site 3	Metro	98	50%	50%	1%	Mainly paper	0	3/7	17/23	13/38
Site 4	Metro	300	75%	21%	4%	Nearly paperless	5	16/20	1/1	30/48
Total							9	23/34	21/28	66/147

#### Recording information

Participants were asked about the amount of documentation they completed/undertook and to estimate the time this consumed for: a) forms filled out to record information during a routine shift; b) incident reporting; and c) medication documentation. Participants were asked whether they used paper or computer systems to record this information and whether it involved the use of mobile technologies. If computers were used, participants were asked where they were located in the facility.

### Volume of documentation processing in RACFs

Across facilities respondents reported that they completed a median of six (range 1–50, mean = 9.6) forms each during a shift (Table 3). Staff from the facility that mainly used paper documentation systems filled out twice as many forms per shift (median number of forms = 11, mean =14.3) as staff from facilities with mostly computer (median = 5, mean = 9.1) and nearly paperless (median = 6, mean = 6.9) documentation systems.

**Table 3:** Amount of forms completed and time spent completing forms per shift

		**Median number of forms completed per shift Mean (range)**	**Median time spent completing forms per shift (minutes) Mean (range)**
	RNs	6	60
		10.7	107.4
		(2.5-35)	(30–36)
		N = 21/23	N = 23/23
Staff type	ENs and AINs	6	30
		9.2	44.2
		(1–50)	(2–300)
		N = 85/87	N = 83/87
	Managers and HIOs	9	360
		10.9	272.5
		(2–24)	(20–390)
		N = 8/9	N = 8/9
	Mainly Paper	11	30
		14.3	54.7
		(4–50)	(10–215)
		N = 32/33	N = 33/34
Facility documentation system	Mostly computers	5	30
		9.1	84.7
		(3–30)	(10–390)
		N = 31/34	N = 33/34
	Nearly paperless	6	37.5
		6.9	77.4
		(1–24)	(2–360)
		N = 51/52	N = 48/52
		6	30
Total - all participants		9.6	73.0
		(1–50)	(2–390)
		N = 114/119	N = 114/119

### Time spent on documentation processing and preparing facility-wide incident reports

Across the facilities the estimated time spent filling out forms per shift ranged from 2 min to 6.5 h, the median time was 30 min (mean =73.0 min) (Table 3). RNs spent twice as long as ENs and AINs filling out forms per shift. Managers and HIOs spent six times longer on filling out forms than RNs. There was little difference in the time spent on this task between different facilities.

Participants were asked how long they spent completing facility-wide incident reports. Across all facilities, 44 staff answered that they performed this task. Twenty-nine (65.9%) responded that they spent less than 30 min performing this task in an average shift; 8 respondents (18.2%) spent 1 h, 5 (11.4%) a few hours and 2 (4.5%) an entire shift.

### Documentation system used at RACFs

Participants were asked whether they used paper, electronic or hybrid (paper and electronic) formats for documentation tasks. Of the 114 respondents, 72 (63.2%), reported that they used hybrid systems (Table 4). RNs were more likely to use electronic only systems for documentation, which was consistent with the finding that RNs spent a median time of 1h 30min per shift (range 35 min - 7 h 30 min, mean = 124.9 min) using a computer/IT.

**Table 4:** Percentage of respondents who used paper, electronic or hybrid formats for recording information

		**Format for recording information**		
		**Paper Only**	**Electronic Only**	**Hybrid**
	Managers and HIOs N = 7/9	0.0%	0.0%	100.0%
Staff type	RNs N = 22/23	9.1%	31.8%	59.1%
	ENs and AINs N = 85/87	36.5%	2.4%	61.2%
	Mainly paper N = 33/33	81.8%	0.0%	18.2%
Facility documentation system	Mostly computers N = 31/34	9.7%	3.2%	87.1%
	Nearly paperless N = 50/52	6.0%	16.0%	78.0%
Total - all participants	N = 114/119	28.9%	7.9%	63.2%

When ENs and AINs were asked how long they spent per shift on a computer/IT it was found they spent the least amount of time (30 min; range 0 min to 8 h a shift, mean = 50.4 min). Managers and HIOs spent the most time (median 6 h; range 3 – 8 h per shift, mean = 356.7 min). Across all facilities the median time each staff member spent using a computer/IT was 45 min (range 0–480 min per shift, mean = 94.5 min).

### Documentation system used for medication management

Participants were asked whether they used paper only, electronic only or hybrid methods of documentation for medication management. Across all facilities, all 48 staff who checked drug expiry dates did so using paper documentation systems; of the 49 staff who checked the correct storage of drugs, 46 (93.9%) did so using paper only; of the 59 staff involved in medication administration, 55 (93.2%) used paper only; out of the 47 staff involved in auditing medications, 42 (89.4%) used paper only; and of the 57 staff involved in medication ordering, 50 (87.7%) used paper only documentation systems for this activity. The remaining respondents for each question indicated that they used hybrid methods for each of the medication activities listed above, except for one person (2%) who responded that they used electronic only methods for checking the correct storage of drugs.

### When information is documented

Respondents were asked when they recorded information about a resident’s care. Respondents could select from: i) at the point-of-care; ii) whenever I get the opportunity to do so during my shift; or iii) at the end of the shift. Of the 91 staff who responded to this question, 52 (57.1%) indicated that they record information “whenever I get the opportunity to do so”. Of the 19 RNs that answered this question, 14 (73.7%) selected that they record information whenever they get the opportunity, 4 (21.1%) responded that they completed documentation at the point-of-care, and the remaining RN responded that they completed documentation at the end of the shift. Of the 68 ENs and AINs who answered this question, 35 (51.5%) selected that they record information whenever they get the opportunity, 7 (10.3%) responded that they complete documentation at the point of care and 26 (38.2%) answered that they recorded information at the end of the shift. The type of documentation system a facility had i.e. mainly paper, mostly computer or nearly paperless, was not associated with when staff entered information into the system. The same trend was revealed across all facilities; thus of the 91 respondents, 52 (57.1%) recorded information whenever they got the opportunity, 27 (29.7%) recorded information at “the end of the shift” and 12 (13.2%) recorded information at “the point-of-care”.

### Locations of computers and use of mobile technology in the facility

Respondents were asked how frequently they used computers at a nurses’ station, in the hallways, at a resident’s bedside or on the medication cart. Respondents could choose from extensively used, often used, sometimes used, rarely used or not used. Of the 102 respondents who had computers available at a nurses’ station, 74 (72.6%) reported using them often or extensively, 13 (12.7%) sometimes or rarely and 15 (14.7%) did not use them at all. Of the 37 respondents who had computers available in the hallways, 15 (40.5%) often/extensively used them, 5 (13.5%) sometimes/rarely used them and 17 (45.9%) did not use them. Of the 17 respondents who had computers available at the bedside, 16 (94.1%), did not use them, with the remaining one respondent answering that they rarely used them. Similarly, of the 18 respondents who had computers on a medication cart available to them 17 (94.4%) did not use them, with the remaining one respondent answering that they rarely used them. Other mobile technologies, such as hand held computers, portable devices/laptops, wireless computers, or PDA/palm pilots were also not as frequently used when compared to the use of computers at the nursing station or in the hallways; of the 30 respondents who had this technology available to them, 5(16.6%) often/extensively used these mobile technologies, 11 (36.7%) rarely/sometimes used them and 14 (46.7%) never used them.

#### Exchange of information

Participants were asked about how information moved through the facility either between paper and electronic documentation systems or between staff members. Information exchange between the facility and external providers such as the GP or community pharmacist was explored in terms of the frequency of the method used and the time this took.

### Transfer of information from paper to electronic systems

Respondents were asked whether they transferred information from paper to computer and if so for what purposes during a typical shift. Respondents could select from the following five options: i) never transfer information from paper to computer; Transcribe for the purpose of ii) clinical documentation; iii) administration; iv) accreditation, or v) funding. Of the 109 respondents, 75 (68.8%) answered that they transcribed information from paper to computer during a typical shift for one or more purposes. The remaining 34 (31.2%) selected that they never transferred information from paper to computer. The most frequent reason for transcribing information from paper to computer was for clinical documentation (72%), followed by funding reasons (60%), administration (34.7%), and accreditation purposes (28%). When asked how long this transcription took, respondents (n = 68) estimated a median time of 30 min spent per shift (range 5 min - 4 h per shift; mean =42.4 min). Managers and HIOs (n = 7) spent the largest amount of time transcribing information from paper to computer, taking a median of 60 min (range 30 min - 2 h a shift; mean =68.6 min). RNs (n = 16) spent 30 min (range 10 min – 2 h; mean = 46.7 min) and ENs and AINs (n = 45) spent 20 min (range 5 min - 4 h a shift; mean =36.7 min).

### Methods used to communicate about a resident’s care needs between staff within the facility

Participants were asked how frequently (never, sometimes, often, always) they used different types of written, verbal or electronic communication to exchange information about a resident’s care needs with one another. Verbal communication, particularly communication at handover, was more frequently used than written or electronic communication methods (Table 5). Second to this was handwritten communication including handwritten notes, communication diaries, whiteboards and folders at the nurses’ station. The least frequently used form of communication was electronic communication, which included email and electronic messages. Participants were asked to give reasons as to why they thought the methods of communication were effective. Coding of the open ended responses revealed eight main categories of reasons: 1) clarity; 2) maximum dissemination of information; 3) continuity; 4) immediacy; 5) proof/evidence; 6) that it was more personal; 7) out of habit; and 8) because that method offered the most current information. Clarity and the maximum dissemination of information were the two main reasons that staff preferred face-to-face and handwritten communication. Electronic communication was favoured because it allowed a large volume of information to be disseminated to a large number of people, and email in particular was favoured because it provided proof or evidence of the communication exchange.

**Table 5:** Methods of communication used between staff within the facility

	**Modes of communication about a resident’s care needs**	**Frequency of use**			
		**Never**	**Sometimes**	**Often**	**Always**
	Handover N = 111	0.0%	4.5%	16.2%	79.3%
Verbal communication	Face to Face communication N=113	0.0%	7.1%	39.8%	53.1%
	Phone N = 100	3.0%	31.0%	30.0%	36.0%
	Communication diary N = 83	2.4%	18.1%	33.7%	45.8%
Written communication	Handwritten notes/progress notes N = 97	5.2%	27.8%	23.7%	43.3%
	White board/notice board N = 73	47.9%	24.7%	8.2%	19.2%
	Folder at nurses station N = 88	6.8%	17.0%	27.3%	48.9%
	Email N = 75	29.3%	28.0%	24.0%	18.7%
Electronic communication	Electronic messages N = 80	27.5%	17.5%	17.5%	37.5%

### Medication information exchange external to the facility

Community pharmacies and General Practitioners have an integral role in providing clinical services to RACFs. Community pharmacies supply, dispense and review medications and General Practitioners provide all medical services as RACFs do not have medical doctors on staff. Thus the RACFs rely heavily on the use of telephones and faxes to communicate with these external partners. Respondents reported that during an average shift they sent a median of 2 faxes (mean =2.9) to pharmacies, and sent a median of 1.5 faxes (mean = 2.9) to General Practitioners. Additionally, staff reported initiating a median of 2 phone calls per shift (mean = 2.5) to pharmacies and initiating a median of 1.5 calls per shift (mean = 2.2) to General Practitioners for medication related issues (Table 6). The number of faxes and calls made to external providers were higher for facilities that used more electronic documentation systems (Table 6). Participants were asked how long they spent communicating with pharmacies including all delays such as waiting on the phone about medication orders. Of the 53 respondents 47 (88.7%) spent less than 30 min a shift, and the remaining 6 (11.3%) spent between 30 min to 1 hour a shift communicating with pharmacies. When respondents were asked how long they spent per shift communicating with GPs including all delays, of the 54 who responded, 47 (87%) spent less than 30 min a shift, 2 (3.7%) spent between 30 min an hour a shift, and 5 (9.3%) spent over an hour a shift.

**Table 6:** Amount of faxes sent and calls made to General Practitioners and pharmacists per shift regarding prescription issues

		**Median number of faxes sent to pharmacy during a shift Mean (range)**	**Median number of calls to pharmacy during a shift Mean (range)**	**Median number of faxes sent to GP for all prescription- related issues during a shift Mean (range)**	**Median number of calls to GP for all prescription-related issues during a shift Mean (range)**
	Managers and HIOs	3.5	2.5	0.3	0.0
		2.5	2.5	2.1	0.5
		(0–4)	(0–5)	(0–8)	(0–2)
		N = 3	N = 3	N = 4	N = 4
Staff type	RNs	3.0	2.5	0.3	0.0
		3.5	3.1	3.1	2.8
		(1–7.5)	(0–20)	(0–15)	(0.5-15)
		N = 21	N = 21	N = 22	N = 19
	ENs and AINs	1	1.5	1.5	1.0
		2.5	1.9	2.9	2.0
		(0–20)	(0–10)	(0–10)	(0–25)
		N = 24	N = 22	N = 29	N = 22
	Mainly paper	1.0	1.0	0.0	0.0
		0.9	1.0	2.3	0.6
		(0–3)	(0–3)	(0–9)	(0–2)
		N = 15	N = 13	N = 17	N = 13
Facility documentation System	Mostly computers	2.5	2.0	2.0	1.0
		4.2	2.7	2.8	2.7
		(0–20)	(0–10)	(0–10)	(0–25)
		N = 17	N = 17	N = 21	N = 16
	Nearly paperless	3.0	2.8	2.5	2.0
		3.6	3.5	3.6	3.1
		(1–7.5)	(0–20)	(0.5-15)	(1.5-15)
		N = 16	N = 16	N = 17	N = 16
Total - all participants		2.0	2.0	1.5	1.5
		2.9	2.5	2.9	2.2
		(0–20)	(0–20)	(0–15)	(0–25)
		N = 48	N = 46	N = 55	N = 45

#### Retrieval of information

Participants were asked how they retrieved information at the point-of-care, and how accessible information was regarding a resident’s stay in hospital.

### Accessibility of information at the point-of-care

Participants were asked whether the information they needed when caring for a resident was “never located in a different place, because it was always available at the point-of-care”, if information was “occasionally located in a different place” or if information was “often found in different locations”. Of the 104 respondents, 64 (61.5%) reported that information regarding a resident was more likely to be located in a different place (30 staff reported that information was *occasionally* located in a different place, and 34 staff reported it was *often* located in a different place). The remaining 40 (38.5%) respondents reported that they had the necessary information available at the point-of-care. The availability of information at the point-of-care was associated with the level of IT at each site. Of the 40 respondents who answered that they always had information available at the point-of-care, 16 (40%) came from a facility mainly using paper documentation systems, whereas 14 (35%) came from a facility which mostly used computers, the remaining 10 (25%) came from a nearly paperless facility.

### Accessibility of information regarding residents’ hospital admissions

Respondents were asked how available information was (always, often, sometimes, never) regarding a resident’s stay in hospital. Of the 96 staff who responded, 34 (35.4%) responded they always had access to information about a resident’s hospital stay, 28 (29.2%) responded that they often had access, 25 (26%) that they sometimes had access and 9 (9.4%) staff said they “never” had access. Just over half (54.5%) of the 22 RNs who responded to this question reported that they often had access to this information, 8 (36.4%) answered that they sometimes had access, but only 2 (9.1%) of the RNs reported that they *always* had access to this information. Of the 68 ENs and AINs who responded, 31 (45.6%) reported always having access to this information, 13 (19.1%) reported often having access, 15 (22.1%) reported sometimes having access and 9 (13.2%) reported never having access to this information.

## Discussion

This study used a survey of RACFs to explore information exchange both within RACFs and between the facilities and GPs, community pharmacists and hospitals. The results revealed that information processing is a major staff activity and that information input and exchange were time consuming, especially for RNs. Inefficient and potentially unsafe practices [14] were identified, such as the vast amount of transcribing information from paper to computer systems, a task which was reported by nearly 70% of respondents. Further, the extensive reliance upon faxes as the main conduit for communication between external providers highlights the urgent need for interoperable information systems to facilitate efficient and accurate communication. A concerning finding was that around 30% of respondents reported that they sometimes or never had access to information following a resident’s hospital admission; this is another aspect of communication which could be enhanced by improved information systems use.

Staff completed a high volume of documentation with a median of 6 forms filled out per staff member per shift. This rate nearly doubled for respondents working in a RACF that mainly used paper documentation systems. Documentation was found to be a time consuming process, which confirms previous reports [15-17]. This was particularly the case for those involved in incident reporting and for RNs who spent more time on documentation than ENs and AINs. This in part confirms the qualitative literature which describes RNs as the information gatekeepers, and ENs and AINs as the hands on workers [18]. This documentation is critical to the care process; however studies have shown that staff believe this time could be better spent attending to residents [3,16-21]. Thus from a staff member perspective, a user friendly documentation system that reduced time spent on documentation- related tasks would be favourable. There is some evidence to suggest that electronic nursing documentation systems encompassing electronic progress notes, care plans, handover sheets, scheduling and funding calculations within facilities reduce the time spent on documentation [22]. Electronic documentation systems could also potentially reduce the time spent at handover as nurses would not have to search for information from different locations as is currently performed [23]. However, prior evidence, as well as the findings from this study, suggest that internal facility electronic documentation systems either make no difference or even increase documentation time [24-26]. These conflicting reports suggest that a greater understanding of the work processes and information exchange requirements of RACFs is needed to inform the design and implementation of IT systems that are efficient, user friendly, and which better support data input related to clinical and care tasks [8].

Results of this study confirm prior findings [12], which have shown that the majority of care is documented at nurses’ stations, away from the point-of-care and whenever staff have the opportunity. The use of mobile technology may assist in reducing the delay in data input, as well as improve the accuracy of data as it would be entered into the electronic system directly (rather than transcribed) thus reducing the potential chance of transfer error or omission of information. Only 40% of respondents indicated that they always had information available at the point-of-care, the majority of whom came from a facility using paper based documentation systems, suggesting that if electronic information systems are to be implemented, they need to be designed so that they are readily accessible at the point-of-care for information retrieval. This could either occur by having appropriate ratios of computers to staff, having computers located where staff document, or having mobile technology that enables documentation at the resident bedside. However surprisingly, we also found that staff who had access to computers at the bedside or on mobile medication carts reported that they rarely used these computers. Previous studies have found that mobile technologies available to clinicians in hospital often do not choose to use them at patients’ bedsides [27]. Considerably more research is required to understand when and what benefits mobile technologies deliver within healthcare settings [28].

Communication with external providers was found to be cumbersome with a high reliance on calls and faxes as a means of communication between RACFs and GPs and pharmacists. Results showed that a median of 2 faxes were sent to pharmacy and 1.5 faxes were sent to General Practitioners per staff member per shift. Additionally, 2 calls were made to pharmacy and 1.5 calls were made to General Practitioners per staff member per shift. Communication with hospitals regarding a resident’s stay in hospital was found to be lacking, with only a third of staff always having access to information related to a resident’s recent hospital stay. A potential explanation as to why the majority of nursing staff in our study didn’t always have access to this information could be that the RNs at an RACF receive and process incoming information from the hospital then filter it down to ENs and AINs in a different format. Other studies have also identified this lack of communication between hospitals and RACFs; McCloskey’s ethnographic study in one nursing home and one Emergency Department (ED) in an urban centre in Canada, made observations of hospital-to- nursing home transfer. Even though nursing staff from the ED reported that the ED record was routinely sent back with the residents, the investigator observed few cases of this actually happening, and even when a record was sent to the facility, it contained little information as to what had transpired whilst a resident was in the ED [29]. Unlike internal facility issues, these issues of external communication are more challenging to resolve with IT as it relies on the interoperability between facility and GP or pharmacist or hospital IT systems. National efforts to link up electronically controlled health records via a personally controlled electronic health record, as is envisioned for Australia [30], would greatly facilitate the exchange of information between aged care facilities and other health facilities and practitioners and would aid in reducing the excessive amount of telephone calling and faxing to these providers by RACFs. Additionally, interoperable electronic systems in which health records were accessed by providers could reduce the need for multiple health records of residents/patients, allowing for a more consistent record to be kept. A study conducted by Burns et al. [31], in four RACFs trialling electronic medication charts, found GPs reduced their need for duplicate copies of patients’ notes at the surgery once they could remotely access the electronic medication chart at the RACF. The development of interoperable systems may also overcome the silo effect of communication, as if often described [32], amongst these three entities.

The results revealed that, for internal communication, face-to-face communication was favoured as it allowed staff to clarify issues with one another more easily, and also it was the most effective means of communicating a large amount of information about a resident’s care to colleagues. Studies looking at *handover* communication between nursing staff, found that a combined oral and written communication at handover achieved higher standards of documentation of care, than if verbal communication alone was used. [33]. Thus, while face-to-face communication is a fundamental part of health care provision and appropriately the most central form of communication, it is likely that innovative use of IT could potentially enhance internal communication processes within the RACF, such as handover.

We acknowledge that there are general limitations with using surveys as a research tool [34], including participants’ understanding of the questions as a result of having English as a second language. We did not ascertain whether English was a second language for participants, as the nurse managers in the focus group recommended that inclusion of this question may deter participants who wanted to remain anonymous. Even had we asked whether workers had English as a second language, this still would not have provided us with an indication of English language proficiency. While this study represents one of the few multi-site surveys of RACFs, and the first to provide a detailed examination of information exchange processes, the sample size was modest. Larger scale studies would be valuable to confirm the generalisability of the findings. The nature or purpose of documentation outside incident and medication-related documentation was not ascertained in this survey. This information would be valuable for further investigation in future studies.

## Conclusions

This study has contributed new evidence of information input, exchange and retrieval in RACFs. It is only with knowledge of such processes that solutions can be tailored to reduce inefficient information exchange processes and those that adversely affect the continuum of care for residents. Well designed information systems, particularly those that are interoperable with the systems used by external partners, would greatly facilitate the provision of safe, better-coordinated and better-quality care, while also reducing the burden of information documentation on RACF staff.

## Competing interests

The authors declare that they have no competing interests.

## Authors’ contributions

All authors were involved in the survey analysis and interpretation of the data. AG/JW/DB were involved in the conception design and data collection of the survey. Both SG and AG drafted the paper. All authors were involved in the interpretation of the results and the critical revision of the paper. All authors read and approved the final manuscript.

## Pre-publication history

The pre-publication history for this paper can be accessed here:

http://www.biomedcentral.com/1471-2318/12/40/prepub

## Supplementary Material

Additional file 1 Identifying information work processes in Residential Aged Care Facilities (RACFs) in Australia. Click here for file
