# Supplementary material for: Examining the role of information exchange in residential aged care work practices-a survey of residential aged care facilities
Source: BMC Geriatr. 2012 Aug 2;12:40. doi: 10.1186/1471-2318-12-40 (PMC3463451; doi:10.1186/1471-2318-12-40)
Supplement: Additional file 1 — Identifying information work processes in Residential Aged Care Facilities (RACFs) in Australia. [file 1471-2318-12-40-S1.docx]

**Identifying information work processes in Residential Aged Care Facilities (RACFs) in Australia**

Thank you for agreeing to complete this short survey. We estimate the survey will take 10 to 15 minutes to complete. The information will help to identify where information and communication technology can contribute to improved work process and care provision in residential aged care facilities.

# A. The recording of information in your facility

1. How many different forms would you estimate you use to record information during a routine shift?

different forms

1. For the majority of these forms how is information recorded? (Please tick appropriate box)

On paper only

Electronically only

Both on paper and electronically

1. When do you mostly record information about a resident during your shift? (Please tick appropriate box)

At the point of care

Whenever I get the opportunity to do so during my shift

At the end of the shift

1. Approximately how many minutes/hours would you estimate you spend documenting information during a shift?

minutes/hours

1. During your normal shift are you required to transfer information from paper to computer for any of the following? (Please tick all the categories that apply)

For administrative purposes

For accreditation purposes

For ACFI purposes

For clinical documentation

Not applicable (I never transfer information from paper to computer)

1. How much time during the shift do you spend in transferring information (paper to electronic) during the shift? (Please provide an answer in minutes/hours)

minutes/hours

1. Please circle the answer that best indicates the extent you use each of the following technologies during a single shift at your facility.

|  | Not available | Not used | Rarely used | Sometimes used | Often used | Extensively used |
| --- | --- | --- | --- | --- | --- | --- |
| Computer at the nursing station | NA | 1 | 2 | 3 | 4 | 5 |
| Computer in the hallways | NA | 1 | 2 | 3 | 4 | 5 |
| Computer on the medication cart | NA | 1 | 2 | 3 | 4 | 5 |
| Computer at the bedside | NA | 1 | 2 | 3 | 4 | 5 |
| Portable computing devices used (eg, hand-held computers, portable devices or laptops, wireless computers, PDA/Palm) | NA | 1 | 2 | 3 | 4 | 5 |
| Computer with touch screens | NA | 1 | 2 | 3 | 4 | 5 |

1. Please circle your answer about your level of computing skill:

|  |  | Poor | Fair | Good | Very good | Excellent |
| --- | --- | --- | --- | --- | --- | --- |
|  | I would rate my computing skills as | 1 | 2 | 3 | 4 | 5 |

1. For each statement please circle the answer that best indicates the level of IT/computer support that is available to you at your facility.

|  |  | Strongly disagree | Disagree | Undecided | Agree | Strongly agree |
| --- | --- | --- | --- | --- | --- | --- |
|  | My facility provides a high level of computer instruction and education | 1 | 2 | 3 | 4 | 5 |
|  | A specific person (or group) is available to provide computer assistance in my facility | 1 | 2 | 3 | 4 | 5 |

# B. How is information accessed?

1. When you are caring for a resident, is the information you need located in a different place? (please tick only one box)

Never, information is always available at point of care electronically

Never, information is always available at point of care in the resident’s folder

Occasionally, only when very specific information is required

Often, as relevant information is located in different locations

Not applicable

# C. The management of medications

1. Are you involved in medication procedures for residents at your facility?

Yes No

***If No then please go to Section D (question no. 20)***

1. How are the following medication processes/activities performed within your facility? (Please place a tick in the appropriate box)

| **Activity** | Paper based | Electronic only | Both paper and electronic | Not applicable |
| --- | --- | --- | --- | --- |
| Medication ordering |  |  |  |  |
| Auditing chart for compliance |  |  |  |  |
| Checking drug expiry dates |  |  |  |  |
| Checking for correct storage |  |  |  |  |
| Medication Administration |  |  |  |  |
| Other significant activity (please specify) |  |  |  | |

1. On average (during one shift), how long do you spend communicating with pharmacies (including all delays such as waiting on the phone) about medication orders? (Please tick appropriate category)

0 -29 min per shift

30 min -1hr per shift

More than 1hr per shift

1. On average (during one shift), how many faxes do you send to pharmacies?

faxes sent per shift

1. On average (during one shift), how many phone calls would you have with the pharmacy?

phone calls per shift

1. How long (including all delays) would you spend per shift communicating with GPs about prescription related issues? (Please tick appropriate category)

0-29 min per shift

30 min -1hr per shift

More than 1hr per shift

1. On average how many faxes do you estimate you send to GPs per shift for all prescription-related issues?

faxes per shift

1. On average how many phone calls do you estimate you have with the GPs or practice per shift for all prescription-related issues?

phone calls to GPs per shift

1. On average how many emails or electronic messages do you estimate you exchange with GPs per shift for all prescription related issues?

electronic messages per shift

# D. Preparation of facility and resident-related summary reports

*If you are not involved in the preparation of these reports, please go to Section E (Question 23).*

1. How long does it take you to prepare (collect information and write up) a facility-wide incident analysis report? (Please tick appropriate category)

Hardly any time at all (< 30 minutes)

About an hour

A few hours

A whole shift

Not applicable

1. Do you prepare an incident analysis report using a computer-generated report structure/template? (Please tick appropriate category)

Not applicable - there is no computerised system available

Not at all

Only for some parts of the report

Most parts are computer generated

All the time

1. If you use the computer for preparing an incident analysis report, which computer applications do you use to create these reports? (Please tick appropriate category)

Microsoft Office (includes MS Word, MS Excel & MS PowerPoint)

Specific IT system available in my facility

Other –Please specify

# E. Communicating with staff across the facility

1. How do staff within your facility contact you about a task or job related to a resident’s general care needs? (Please provide an answer for each category by ticking the appropriate box)

|  | **Methods of communication about a resident’s care needs** | **How frequently my facility uses this method** | | | | |
| --- | --- | --- | --- | --- | --- | --- |
|  |  | **Not applicable** | **Never** | **Sometimes** | **Often** | **Always** |
| a) | Hand written notes/Progress notes |  |  |  |  |  |
| b) | Communication diary |  |  |  |  |  |
| c) | Face to face communication |  |  |  |  |  |
| d) | Folder at nurses station |  |  |  |  |  |
| e) | Phone |  |  |  |  |  |
| f) | Email |  |  |  |  |  |
| g) | White board/notice board |  |  |  |  |  |
| h) | Electronic messages |  |  |  |  |  |
| i) | Handover |  |  |  |  |  |
| j) | Other (please specify) |  | | | | |

1. Which of these methods of communication do you regard as most effective? (Tick appropriate boxes)

Hand written notes Communication diary Face to face

Folders at nurses station Phone Email

White board/notice board Electronic messages Handover Other

Why do you think these methods are effective? Please use the following space to explain your answer further.

# F. Communication with a Resident’s Family

1. How many times in an average shift would you communicate with a resident’s family?

times per shift

1. How long (minutes or hours per shift) in total would you estimate you spend communicating with resident families in a shift?

minutes/hours per shift

1. How often do you use the following methods to communicate with the resident’s family? (Please tick a box for each category)

|  | **Channel used** | **Not applicable** | **Never** | **Sometimes** | **Often** | **Always** |
| --- | --- | --- | --- | --- | --- | --- |
| a) | Face to face communication |  |  |  |  |  |
| b) | Fax |  |  |  |  |  |
| c) | Phone |  |  |  |  |  |
| d) | Email |  |  |  |  |  |
| e) | Letters (posted mail) |  |  |  |  |  |
| f) | Electronic messages (SMS/voice messages) |  |  |  |  |  |
| g) | Tele-Conferencing (Skype) |  |  |  |  |  |
| h) | Other (please specify below) |  |  |  |  |  |

1. Which methods do you regard as the most effective way of communicating a resident’s family? (Please tick )

Face to face communication Fax Phone

Email Letters (posted mail) Electronic messages

Teleconferencing (Skype) Other

Why do you think these methods are the most effective? Please use the following space to explain your answer further.

1. What would you say are the three most common issues/reasons about which you communicate with the resident’s family?

| 1. |
| --- |
| 2. |
| 3. |

**G. Communication with people outside the facility**

1. When a resident is transferred from hospital, how often is information about what happened to the resident while in hospital available?

Always

Often

Sometimes

Never

Not applicable

# H. The use of information and communication technologies in your facility

1. In general how much of your time during a shift would you estimate involves use of a computer or information technology? Please provide answer in minutes/hours.

minutes/hours per shift

# Demographics

1. What is your job title?
2. Age (please tick appropriate box)

Less than 20

20–29

30–39

40–49

50 or over

1. How many years have you worked (either here or elsewhere) in your current position?
2. What is your highest level of education? (Please tick appropriate category)

Secondary school

Post secondary (technical)

Tertiary education

**You have reached the end of the survey.**

Thank you very much for completing the survey. A summary of the results will be provided to participating sites.
